# Analysis of Long-Term Cochlear Implantation Outcomes and Correlation With Imaging Characteristics in Patients With Common Cavity Deformity

**DOI:** 10.3389/fnins.2022.857855

**Published:** 2022-03-23

**Authors:** Lifang Zhang, Biao Chen, Ying Kong, Natalia Liau, Xingmei Wei, Ying Shi, Jingyuan Chen, Mengge Yang, Anandhan Dhanasingh, Yongxin Li

**Affiliations:** ^1^Department of Otolaryngology, Head and Neck Surgery, Beijing Tongren Hospital, Capital Medical University, Beijing, China; ^2^Key Laboratory of Otolaryngology, Head and Neck Surgery, Ministry of Education, Capital Medical University, Beijing, China; ^3^Beijing Institute of Otolaryngology, Ministry of Education, Capital Medical University, Beijing, China; ^4^MED-EL Medical Electronics GmbH, Innsbruck, Austria

**Keywords:** common cavity deformity, cochlear implantation, auditory development, speech development, 3D reconstruction

## Abstract

**Object:**

To investigate the long-term development of auditory and speech in patients with common cavity deformity (CCD) after cochlear implantation (CI) and its relationship to imaging characteristics.

**Methods:**

Twenty-three CCD patients and 59 age- and sex-matched CI children with normal inner ear structure were recruited. The auditory and speech development of these two groups were evaluated at 0, 1, 3, 6, 12, and 18 months after CI activation using four parent reports questionnaires [Categories of Auditory Performance (CAP), Speech Intelligibility Rating (SIR), Meaningful Auditory Integration Scale/Infant-Toddler Meaningful Auditory Integration Scale (MAIS/ITMAIS), and Meaningful Use of Speech Scale (MUSS)]. Computed tomography-based 3-dimensional reconstruction of the surgical side of 18 CCD children was performed, the volume and surface area were calculated. Correlation analysis was performed on the imaging performance and post-operative outcomes.

**Results:**

The percentages of MAIS/IT-MAIS scores and CAP scores at different evaluation time points are significantly different (*p* < 0.05). When comparing SIR results across time points, significant growth was observed in most of the comparisons. In addition, significant differences (*p* < 0.05) are observed among the percentages of MUSS scores at different time points except the comparison between 0 and 1 month after CI activation. Patients in the CCD group had poorer auditory and speech performances at different stages after CI compared with those in the control group. According to the reconstruction of CCD patients, the volume ranged from 12.21 to 291.96 mm^3^; the surface area ranged from 27.81 to 284.7 mm^2^. When the lumen surface area was <190.45 mm^2^ or the volume was <157.91 mm^3^, the survival time for CCD children to achieve a CAP score of 4 after CI was significantly shorter.

**Conclusion:**

Cochlear implantation are less effective in CCD patients than in patients with normal inner ear structures, but they can still achieve significant improvement post-operatively. The morphology and size of the inner ear vary in CCD patients, which reflects the degree of inner ear development influences the outcome after CI surgery.

## Introduction

Common cavity deformity (CCD) is characterized by the presence of an abnormally ovoid or round chamber formed by the cochlea and vestibule and is generally associated with profound sensorineural hearing loss (SNHL) (Sennaroğlu and Bajin, 2017). This condition occurs due to the arrest of otocyst development during the fourth week of embryonic development (Jackler et al., 1987). CCD is diagnosed primarily on the basis of computed tomography (CT) or magnetic resonance imaging (MRI), showing a single fluid-filled cavity of the cochlea and vestibule (Casselman et al., 2001). This malformation varies in size and shape as well as in the location of the internal auditory canal and its size is often assumed to be related to the arrest time of the cochlear vestibule development; that is, the larger the cystic cavity, the later the arrest (Brotto et al., 2019).

For CCD patients, we often choose a cochlear implant (CI) or an auditory brainstem implant (ABI) to help them restore their hearing. Since the ABI was first used in a patient with neurofibromatosis type 2 in 1979, its indications have been constantly updated. In recent years, ABI has been applied to patients with profound SNHL who suffer from conditions such as cochlear sclerosis and severe cochlear malformations (Colletti et al., 2005; Bozorg Grayeli et al., 2007; Sennaroğlu et al., 2016). Sennaroğlu et al. (2016) performed ABI surgeries on seven patients with CCD and found that these patients achieved better hearing threshold and language outcome scores compared with patients with other types of severe cochlear malformations. Nevertheless, given the complications following ABI, such as cerebrospinal fluid leakage, electrode displacement, and limited post-operative benefit (Toh and Luxford, 2008), cochlear implantation (CI) is currently the primary intervention for CCD.

In 1986, McElveen et al. (1997) reported the first CI in a patient with CCD using the transmastoid facial recess approach. Since then, an increasing number of CCD patients have received CI surgery, and the surgical approach and electrodes have continually improved (Molter et al., 1993; Tucci et al., 1995; McElveen et al., 1997; Beltrame et al., 2000, 2005, 2013; Sennaroglu et al., 2014). In 2017, our team proposed that custom-made electrodes could be implanted *via* the transmastoid slotted labyrinthotomy approach (TSLA) for CCD patients (Wei et al., 2018). Instead of conventional electrodes, we used custom electrodes made by MED-EL, with 12 electrodes in the middle of the electrode array and extension wires made of inert silicone carriers containing platinum wires. This strategy allows the electrodes to remain as attached to the lumen as possible, allowing them to stimulate a larger area, which may mean that more spiral ganglion cells are stimulated, resulting in better post-operative outcomes.

Common cavity deformity patients could have long-term benefits with CI (Al-mahboob et al., 2022). Although most studies have concluded that post-operative outcomes in CCD patients are worse than in children with normal inner ear structures and mild malformations, specific auditory speech rehabilitation outcomes are inconsistent (Ahmad et al., 2005; Papsin, 2005; Ahn et al., 2011). In addition, a previous study showed a correlation between the effect of CI implantation and imaging performance (Wei et al., 2017). However, to date, no study has investigated the relationship between post-operative CI outcomes and imaging performance in CCD patients. The present study aims to understand the post-operative auditory-speech performance and developmental patterns of the CCD children implanted with custom electrodes *via* the TSLA approach by analyzing the long-term post-operative outcomes of these children and their relationship with imaging characteristics.

## Materials and Methods

### Participants

A total of 23 pediatric patients (12 female and 11 male) with CCD were recruited from April 2016 to January 2020 at the Department of Otolaryngology Head and Neck Surgery of our hospital. High-resolution computed tomography (HRCT) and inner ear MRI were performed before surgery in all cases, and the diagnosis of CCD was confirmed by two or more physicians from the Radiology and Otorhinolaryngology departments.

Inclusion criteria were as follows.

(1) Bilateral severe or profound SNHL patients diagnosed with CCD.
(2) Available for post-operative follow-up.

Exclusion criteria were as follows.

(1) Patients with contraindications to cochlear implantation.
(2) Patients with history of serious systemic diseases or intellectual disorders.

The age at implantation ranged from 0 to 7 years (mean: 27.65 months, standard deviation: 13.79 months).

A total of 59 congenitally severe or profound SNHL children who met the inclusion criteria and who had normal inner ear structure, matched for age and sex, were recruited as a control group. The age range was 0–8 years (mean, 29.00 months; standard deviation, 20.14 months).

In compliance with ethical standards for human subjects, written informed consent was obtained from the guardians of all participants before proceeding with the study procedures. This study was approved by the Institutional Review Board of our hospital.

### Procedures

#### Routine Clinical Investigations

All participants underwent routine otorhinolaryngological examination, followed by audiological tests, CT, and MRI scans before the CI surgery. To investigate the audiological status in terms of hearing level, function of the central auditory system, and the development of the auditory system, the following audiological tests were performed: (1) Behavioral hearing assessment, (2) Auditory Steady State Response, (3) Auditory Brainstem Response, (4) Distorted Product Otoacoustic Emissions, (5) conventional low 226 Hz tympanometry, and (6) 40-Hz auditory-evoked related potential.

#### Surgical Approach

In the CCD group, all children were implanted with customized electrodes using a transmastoid slotted labyrinthotomy approach (TSLA) (Wei et al., 2018). In the TSLA, the bony wall of the cavity was exposed after mastoidectomy, and a slot was made in the area where the lateral semicircular canal is commonly situated away from the facial nerve. A customized electrode was placed in the cavity toward the cochlear side after the perilymph flow abated (Shi et al., 2019). The electrodes were fully implanted in all children except for one child who had two extra cochlear electrodes because of the small size of the common cavity. None of the children had post-operative complications, such as facial paralysis or cerebrospinal fluid leakage.

In the control group, electrodes were implanted using the conventional transmastoid facial recess approach. All electrodes were successfully implanted.

#### Post-operative Follow-Up Questionnaires

The assessment of the child’s auditory and speech development was performed using the categories of auditory performance (CAP), Speech Intelligibility Rating (SIR), Meaningful Auditory Integration Scale/Infant-Toddler Meaningful Auditory Integration Scale (MAIS/ITMAIS), and Meaningful Use of Speech Scale (MUSS). The parents or guardians of the infants were asked to complete the questionnaires. The questionnaires were evaluated at the activation of CI and at 1, 3, 6, 9, 12, 18, 24, and 36 months after activation. For the MAIS/IT-MAIS and MUSS, we converted the actual scores into percentages as final statistics, and the result was expressed as a percentage using the following equation: total score/40 × 100%.

#### Imaging Evaluation

All patients underwent a temporal bone CT scan (GE 64-row helical CT, Boston, MA, United States) and inner ear MRI (Philips Ingenia 3.0 T MRI scan, Amsterdam, Netherlands) at our hospital before surgery. The E-3D digital medical design system (Liao, 2018) was applied to reconstruct the lumen on the CI side in the CCD group to obtain the volume and surface area of the cavity.

### Data Analysis

Statistical analyses were performed using SPSS 22.0. The results of the MAIS/IT-MAIS and MUSS for both groups did not comply with the normal distribution by the Shapiro-Wilk test. The results of the non-normal distribution were described using the median (25th percentile, 75th percentile) and a non-parametric test was used to compare whether their differences were significant between the two groups and between the various assessment stages. Since the results of the cap questionnaire as well as the SIR questionnaire were rank data, the Wilcoxon test was used to compare whether there was a significant difference between the results of the two groups and between the various assessment stages. For lumen volume and surface area, Kaplan-Meier survival analysis was used to analyze their correlation with post-operative outcomes. Considering that patients with a CAP score of 4 could discriminate speech sounds without the aid of lip-reading, we defined a CAP score of 4 as the endpoint event. The post-operative time for a child to reach a CAP score of 4 was defined as the survival time. The median volume and median surface area were used as criteria for grouping. At the same time, *p* < 0.05 was considered a statistically significant difference.

## Results

**• Demographic information of the CCD group**

Table 1 summarizes the subject information, including gender, age at implantation, and length of follow-up. In the CCD group, there were 11 males and 12 females, with an implantation age of 0–7 years and a mean implantation age of 28 months, while the control group consisted of 31 males and 28 females, with an implantation age of 0–8 years and a mean implantation age of 29 months. The groups were not statistically different in terms of age and gender after non-parametric testing (*p < 0.05*).

**TABLE 1:** Demographic information of the recruited patients.

Group	Gender		Mean implantation age (x̄ ± SD)	Average length of follow-up (M)	Maximum length of follow-up (M)
	Male	Female			
CCD	11	12	27.65 ± 17.30	23	48
Control group	31	28	29.00 ± 20.41	28	48

• **Development of auditory ability in the CCD Group**

As shown in Tables 2, 3, both the percentage of MAIS/IT-MAIS scores and the CAP scores were significantly different between each time point in the CCD group (*p* < 0.05).

**TABLE 2:** Comparison of the percentage of Meaningful Auditory Integration Scale/Infant-Toddler Meaningful Auditory Integration Scale (MAIS/IT-MAIS) scores for each time point in the common cavity deformity (CCD) group.

	0 m	1 m	3 m	6 m	12 m
1 m	0.012				
3 m	0.018	0.005			
6 m	0.018	0.008	0.036		
12 m	0.008	0.003	0.010	0.008	
18 m	0.043	0.043	0.012	0.025	0.027

**TABLE 3:** Comparison of the categories of auditory performance (CAP) scores for each time point in the common cavity deformity (CCD) group.

	0 m	1 m	3 m	6 m	12 m
1 m	0.011				
3 m	0.007	0.034			
6 m	0.003	0.005	0.016		
12 m	0.002	0.002	0.001	0.015	
18 m	0.014	0.007	0.003	0.011	0.015

• **Development of speech in the CCD Group**

For the percentage of MUSS scores (Table 4), there was no significant difference between the results of each time point except between 0 and 1 month after cochlear activation in the CCD group (*p* < 0.05). The SIR scores of the CCD group (Table 5) were significantly different between 0 and 6 months, 1 and 6 months, 0 and 12 months, 1 and 12 months, 3 and 12 months, 6 and 12 months, 0 and 18 months, 1 and 18 months, 3 and 18 months, and 6 and 18 months after cochlear activation (*p* < 0.05).

**TABLE 4:** Comparison of the percentage of Meaningful Use of Speech Scale (MUSS) scores for each time point in the common cavity deformity (CCD) group.

	0 m	1 m	3 m	6 m	12 m
1 m	0.068				
3 m	0.005	0.016			
6 m	0.002	0.012	0.011		
12 m	0.001	0.003	0.002	0.013	
18 m	0.018	0.027	0.017	0.018	0.026

**TABLE 5:** Comparison of the Speech Intelligibility Rating (SIR) scores for each time point in the common cavity deformity (CCD) group.

	0 m	1 m	3 m	6 m	12 m
1 m	1.000				
3 m	0.157	0.157			
6 m	0.046	0.046	0.317		
12 m	0.001	0.001	0.003	0.014	
18 m	0.006	0.006	0.005	0.030	0.257

• **Characteristics of IT-MAIS/MAIS in the CCD and control groups**

**FIGURE 1:**
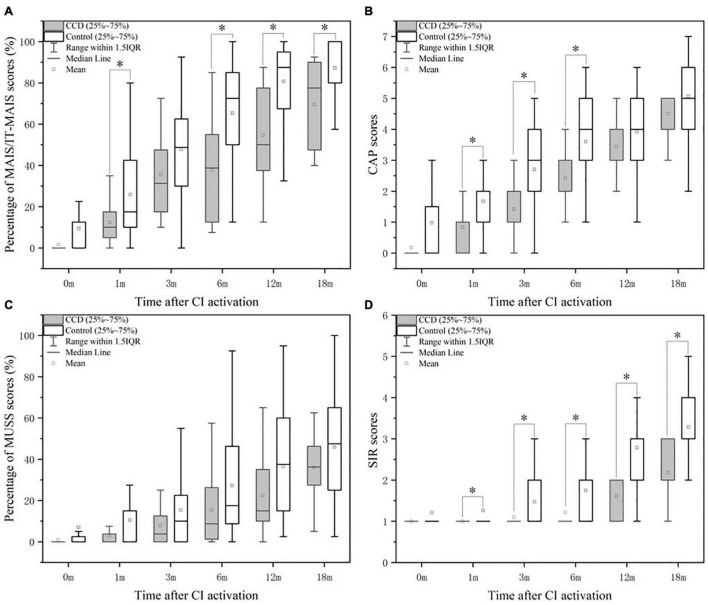
Comparison of the performance in MAIS/IT-MAIS **(A)**, CAP **(B)**, MUSS **(C)**, and SIR **(D)** between the CCD and control groups. CAP, Categories of auditory performance; CCD, common cavity deformity; MAIS/IT-MAIS, Meaningful Auditory Integration Scale/Infant-Toddler Meaningful Auditory Integration Scale; MUSS, Meaningful Use of Speech Scale; SIR, Speech Intelligibility Rating. *indicates significant (*p* < 0.05).

Figure 1A shows the mean, median, and 25th and 75th percentiles of the MAIS/ITMAIS score percentages for the CCD and control groups. The median scores were significantly different between the two groups at 1, 6, 12, and 18 months after cochlear activation (*p* < 0.05). Furthermore, trend of the average MAIS/ITMAIS score percentages of the two groups as the follow-up time increased.

• **Characteristics of CAP in the CCD and control groups**

Similar results were found in the comparison of CAP scores. Significant differences between the two groups were observed at 1, 3, and 6 months after CI activation (Figure 1B). The average CAP scores of the two groups increased over the follow-up period, but the average scores of the control group were higher than those of the CCD group.

• **Characteristics of MUSS in the CCD and Control Groups**

As for the median percentage of MUSS scores, there was no significant difference between the two groups at each time point after CI activation (Figure 1C). Nevertheless, the average percentage of MUSS scores increased slowly over time in both groups, and the CCD group obtained lower scores than the normal group (Figure 1C).

• **Characteristics of SIR in the CCD and control groups**

After statistically analyzing the differences in median SIR scores between the CCD and control groups, we observed that the differences were significant at 1, 3, 6, 12, and 18 months after CI activation (Figure 1D). Similar to the other questionnaires, the mean SIR score in the CCD group increased gradually over time and was worse than that in the control group.

• **Correlations between Imaging Characteristics and CAP results**

**FIGURE 2:**
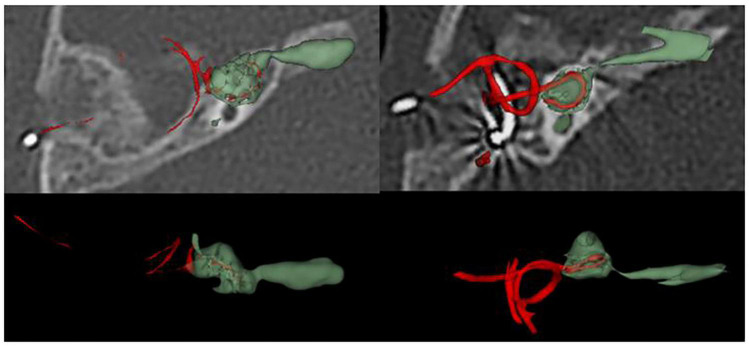
Computed tomography (CT) reconstruction of surgical side in patients with common cavity deformity (CCD).

**FIGURE 3:**
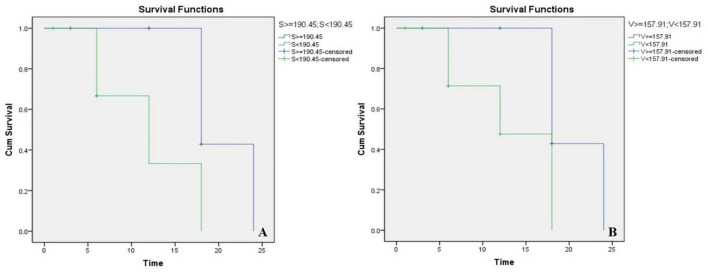
Kaplan-Meier survival chart of different lumen surface areas **(A)** and volumes **(B)**. S, surface areas; V, volumes.

As shown in Figure 2, we reconstructed CT images of the surgical side of the cavity in 18 CCD patients, calculated their volume and surface area (Table 6), and performed a Kaplan-Meier survival analysis with the CAP results (Figure 3). Considering that patients with a CAP score of 4 could discriminate speech sounds without the aid of lip-reading, we defined a CAP score of 4 as the endpoint event. The post-operative time for a child to reach a CAP score of 4 was defined as the survival time. When the lumen surface area was ≥190.45 mm^2^, the mean survival time for CCD children to achieve a CAP score of 4 after surgery was 20.57 months, and the median survival time was 18.00 months; when the lumen surface area was <190.45 mm^2^, the mean survival time for CCD children to reach a CAP score of 4 after surgery was 12 months, and the median survival time was 12.00 months, with a statistically significant difference between the two groups (*p* = 0.02) (Figure 3A). When the lumen volume was ≥157.91 mm^3^, the mean survival time was 20.571 months, and the median time was 18.00 months; when it was <157.91 mm^3^, the mean and median survival times were 13.142 and 12.00 months, respectively. There was a significant difference between the two groups (*p* = 0.022) (Figure 3B).

**TABLE 6:** Surface area and volume after three-dimensional reconstruction of the lumen in common cavity deformity (CCD) patients.

Case	CI side	Surface area (mm^2^)	Volume (mm^3^)
1	Left	224.80	213.96
2	Right	238.00	225.9
3	Left	145.60	128.22
4	Left	284.70	283.6
5	Right	155.60	106.1
6	Left	141.10	117.49
7	Left	238.00	225.9
8	Left	187.10	160.48
9	Right	193.80	182.78
10	Left	254.70	285.07
11	Left	226.90	155.34
12	Left	260.10	291.96
13	Right	97.42	77.77
14	Right	115.90	97.25
15	Left	138.30	113.07
16	Right	224.20	232.94
17	Right	67.85	41.51
18	Right	27.81	12.21

## Discussion

Common cavity deformity is regarded as a severe inner ear malformation, and the post-operative outcome of CI is generally considered to be worse in patients with CCD than in children with normal cochlear structures. In this study, 23 children with CCD were followed up using IT-MAIS/MAIS, MUSS, CAP, and SIR to assess their post-operative outcomes and correlations with imaging performance.

The MAIS is a common tool used to assess functional hearing in hearing-impaired children (Robbins et al., 1991). Each child was assessed by the answers provided by a parent or guardian familiar with the child’s condition. It consists of 10 questions that assess the use of hearing aids and the ability to perceive and understand sounds. The CAP is a scale used to assess the auditory ability of pediatric cochlear implant users in their daily lives (Archbold et al., 1998). The above two questionnaires are effective for evaluating the development of auditory stimuli in children who received cochlear implants or hearing aids. In relation to this, stimulation of the remaining spiral ganglion cells in the cochlea has been found to activate hearing (Fayad and Linthicum, 2006) and it has been suggested that spiral ganglion cells are distributed in the cavity walls of patients with CCD. This is the foundation for hearing acquisition after CI in CCD patients (Brotto et al., 2019). ITMAIS/MAIS and CAP scores were lower in the CCD group than in the normal group after activation. Similar results were reported by Xia et al. (2015). According to Tables 2, 3, the auditory development of CCD patients continuously improved up to 18 months after activation; that is, in the CCD group, there was no significant platform phase of auditory development in the 18 months after the activation of CI. Ahn et al. (2011) also observed an increase in the auditory performance of CCD patients with prolonged follow-up after 48 months of post-cochlear surgery evaluation, with a mean percentage MAIS score of 90.3 ± 18.1% and a mean CAP score of 4.9 ± 1.6. Although auditory development after CI is slower in CCD patients than in CI patients with normal cochlear structures, progress has been consistently made, suggesting the need for long-term post-operative rehabilitation in these patients.

For the assessment of speech development, MUSS and SIR are commonly used instruments. As shown in Tables 4, 5, there were significant differences in both the percentage of MUSS score and the SIR score when comparing the assessment results at different time points. However, the SIR score did not show a significant difference compared to the previous assessment results until 1 year after activation, whereas the percentage of MUSS score showed a significant difference at 3 months after activation, indicating that CCD patients showed a faster increase in MUSS performance compared to SIR. This may be related to the various aspects of speech development assessed by the two questionnaires. Like the MAIS, the MUSS is a parental report scale. It assesses the use of speech and consists of 10 questions designed to evaluate three aspects of speech development: vocalizing behavior, oral communication skills, and oral clarification skills (Archbold et al., 1998; Fayad and Linthicum, 2006). The SIR has been regularly used to evaluate the intelligibility of spontaneous speech in patients with cochlear implants (Xia et al., 2015). That is, the MUSS questionnaire evaluates speech skills, while SIR rates the intelligibility of pronunciation. However, the young age of the children in our study, with a follow-up period of only 1.5 years, made it difficult to demonstrate significant improvements in speech intelligibility. Nevertheless, since the MUSS includes an evaluation of vocalizing behavior, the children were given the opportunity to improve their scores. Additionally, this study observed that the percentage of MUSS scores in children with CCD was higher at 18 months than at 12 months after the activation, and the difference was significant, indicating that speech ability was still improving at 1.5 year after surgery. Xia et al. (2015) also observed a sustained increase in SIR scores for 4 years after CI. Therefore, post-operative speech development in patients with CCD is slow and requires long-term rehabilitation training.

Based on the comparison between the CCD group and the control group, it is obvious that auditory and speech development after CI was poorer in the CCD group than in the CI children with normal cochlear structures. This may be related to the structure malformation, lack of sufficient spiral ganglion cells (Brotto et al., 2019), or developmental delays (Buchman et al., 2004).

However, we observed good outcomes in some children. By 18 months after the activation, 66.67% of children with CCD had a CAP score of 5, but 16.67% achieved a score of only 3. Among the four children with CCD who had been followed for 3 years, only one reached a score of 40 on the IT-MAIS at 3 years after CI activation. We suspect that this may be related to the distribution and number of spiral ganglion cells in the cavity and the location of the electrode. The higher the number of spiral ganglion cells the electrode can stimulate, the greater the benefit to the child.

Furthermore, the post-operative outcome of CCD patients is also related to cerebrospinal fluid gusher, partial electrode insertion, and fewer active electrodes and the contact of the electrode with the inner wall (Dettman et al., 2011; Bae et al., 2022). The present study explored the relationship of post-operative outcomes with the degree of inner ear development in these patients.

With the development of imaging technology, techniques to assess the development of inner ear structures have become more sophisticated (Skinner et al., 2002; Verbist et al., 2010). After evaluating 36 cochleae with inner ear malformations using volume-rendering technique reconstruction and MPR, Ma et al. (2008) concluded that cochlear development could be more clearly assessed using volume-rendering technique reconstruction and MPR. We calculated the lumen volume and the surface area of 18 CCD patients using three-dimensional reconstruction techniques. The volume ranged from 12.21 to 291.96 mm^3^, with a mean volume of 163.98 ± 84.02 mm^3^; the surface area ranged from 27.81 to 284.7 mm^2^, with a mean value of 178.99 ± 71.85 mm^2^. This result implies that patients with CCD have variable cochlear morphology and size differences, which is consistent with the findings of Dhanasingh et al. (2019). This difference may require serious consideration of the surgical approach and the choice of electrodes.

Furthermore, after analyzing the correlations between volume, surface area, and post-operative outcomes of CCD patients, we found that the smaller the lumen, the shorter the time to reach a 4-point post-operative CAP. We speculate that this may be due to the smaller lumen, whose spiral ganglion cells may be more densely distributed, thus providing a larger effective area of electrode stimulation, which results in a greater likelihood of stimulation to ganglion cells. Earlier achievement of 4 points in CAP indicates a faster speed of rehabilitation within a year and a half after surgery but is not indicative of higher scores in the distant future.

Therefore, further studies using larger sample sizes and longer follow-up periods are needed to explore the distribution of intracochlear spiral ganglion cells in conjunction with the post-operative electrode location. These studies will provide guidance in the selection of treatment strategies for CCD patients.

## Conclusion

The present study investigated the imaging performance and long-term auditory speech outcomes of 23 children with CCD, who were found to have poorer auditory and speech development and slower progress after CI than the control group. However, CCD patients still showed improvement in auditory and speech abilities at 1.5 year after CI; hence, they required long-term rehabilitation. The reconstruction of the temporal bone CT showed that the size, volume, and morphology of the cavity in CCD patients varied widely, and a small lumen size is associated with shorter time needed to reach a 4-point post-operative CAP. Further studies should be conducted to verify these results and clarify the relationship and mechanism using a larger sample of patients with CCD.

## Data Availability Statement

The original contributions presented in the study are included in the article/supplementary material, further inquiries can be directed to the corresponding author.

## Ethics Statement

The studies involving human participants were reviewed and approved by the Ethics Committee of Beijing Tongren Hospital, Capital Medical University. Written informed consent to participate in this study was provided by the participants’ legal guardian/next of kin.

## Author Contributions

LZ and BC performed experiments, acquired and analyzed the data, drafted and revised the manuscript. YK programmed the CI and critically revised the manuscript. NL performed experiments and revised the manuscript. XW and YS conceived and designed the study, revised the manuscript. JC and MY acquired data and revised the manuscript. AD reconstructed the CT image of CCD patients. YL supervised the study, interpreted the result and critically revised the manuscript. All authors contributed to the article and approved the final and submitted version.

## Conflict of Interest

AD was employed by MED-EL Medical Electronics GmbH. The remaining authors declare that the research was conducted in the absence of any commercial or financial relationships that could be construed as a potential conflict of interest.

## Publisher’s Note

All claims expressed in this article are solely those of the authors and do not necessarily represent those of their affiliated organizations, or those of the publisher, the editors and the reviewers. Any product that may be evaluated in this article, or claim that may be made by its manufacturer, is not guaranteed or endorsed by the publisher.
